# Resting-state fMRI data of awake dogs (Canis familiaris) via group-level independent component analysis reveal multiple, spatially distributed resting-state networks

**DOI:** 10.1038/s41598-019-51752-2

**Published:** 2019-10-24

**Authors:** Dóra Szabó, Kálmán Czeibert, Ádám Kettinger, Márta Gácsi, Attila Andics, Ádám Miklósi, Enikő Kubinyi

**Affiliations:** 1 Eötvös Loránd University, Department of Ethology, Budapest, 1117 Hungary; 2 Hungarian Academy of Sciences, Research Centre for Natural Sciences, Budapest, 1117 Hungary; 3 Budapest University of Technology and Economics, Department of Nuclear Techniques, Budapest, 1111 Hungary; 4 MTA-ELTE Comparative Ethology Research Group, Budapest, 1117 Hungary; 5 MTA-ELTE ‘Lendület’ Neuroethology of Communication Research Group, Budapest, 1117 Hungary

**Keywords:** Functional magnetic resonance imaging, Cognitive neuroscience

## Abstract

Resting-state networks are spatially distributed, functionally connected brain regions. Studying these networks gives us information about the large-scale functional organization of the brain and alternations in these networks are considered to play a role in a wide range of neurological conditions and aging. To describe resting-state networks in dogs, we measured 22 awake, unrestrained individuals of both sexes and carried out group-level spatial independent component analysis to explore whole-brain connectivity patterns. In this exploratory study, using resting-state functional magnetic resonance imaging (rs-fMRI), we found several such networks: a network involving prefrontal, anterior cingulate, posterior cingulate and hippocampal regions; sensorimotor (SMN), auditory (AUD), frontal (FRO), cerebellar (CER) and striatal networks. The network containing posterior cingulate regions, similarly to Primates, but unlike previous studies in dogs, showed antero-posterior connectedness with involvement of hippocampal and lateral temporal regions. The results give insight into the resting-state networks of awake animals from a taxon beyond rodents through a non-invasive method.

## Introduction

Resting-state networks (RSNs) are spatially distributed, functionally connected brain regions, characterized by the correlation of the time series of spontaneous, low frequency (0.01–0.1 Hz) fluctuations of the blood-oxygen level dependent (BOLD) signal, usually acquired in the absence of a specific task, and measured via functional magnetic resonance imaging (fMRI). One widely used method to explore these whole-brain connectivity patterns is spatial independent component analysis (ICA), a data driven, model-free method^1^. ICA is appropriate to describe networks in case of a species which brain’s functional characteristics are yet to be determined, as it does not require selection of a priori seed regions. This method attempts to discover statistically independent source signals from the measured observations, using non-linear transformations while looking for spatial independence^2^.

The structure and assumed tasks of RSNs are of high interest as they have the potential to provide information about the brain’s large scale functional organization^1,3^, and alternations in these networks have been found to correspond with various pathologies such as dementia or ADHD^4^. As a result, the number of human rs-fMRI studies grew rapidly in recent years, while only a handful of studies attempted to describe characteristics of RSNs in non-human animal species. To reveal phylogenetic changes and conserved core physiological mechanisms, it is crucial to compare a diverse range of non-human species^5^. To date, resting-state networks were investigated via fMRI in mice^6^, rats^7^, marmosets^8^ macaques^9^, prairie voles^10^, dogs^11^ and ferrets^12^. Sensorimotor networks, such as visual and/or somatosensory networks have been described in most animal resting-state fMRI studies, but separete visual network(s) were only reported in prairie voles^10^, ferrets^12^, and in primates^8^. Salience-like networks so far have been described in rodents^6^ and primates^8^, while fronto-parietal like components have been found so far only in prairie voles^10^ and primates^9^.

Although putative default mode networks have been described in all of the investigated species, the typical antero-posterior connectedness of the human default mode network was only found in superorder Euarchontoglires (primates and rodents), while the corresponding networks in superorder Laurasiatheria (described in ferrets^12^ and dogs^11,13^) were reported to show antero-posterior dissociation. Our goal was to investigate whether applying the currently available methods yield interpretable results with our setup (proof-of-concept) and if so, what kind of spatially distributed resting-state networks are detectable in a larger sample of awake, unrestrained family dogs in a resting-state fMRI setup, following up on previous reports^11,13^.

## Methods

### Subjects

We measured 22 family dogs (*Canis familiaris*) (age 6.41 ± 3.42 years (*mean* ± *SD*), range 2–13 years, 10 females and 12 males, 7 golden retrievers, 5 border collies, 2 English cocker spaniels, 1 Labrador retriever, 1 labradoodle, 2 mongrels, 1 Chinese crested dog, 1 Cairn terrier, 1 Hungarian vizsla, 1 Australian shepherd). Training procedure has been described in detail in a previous study^14^, and was based on individual and social learning using positive reinforcement.

### Experimental procedure

**Figure 1:**
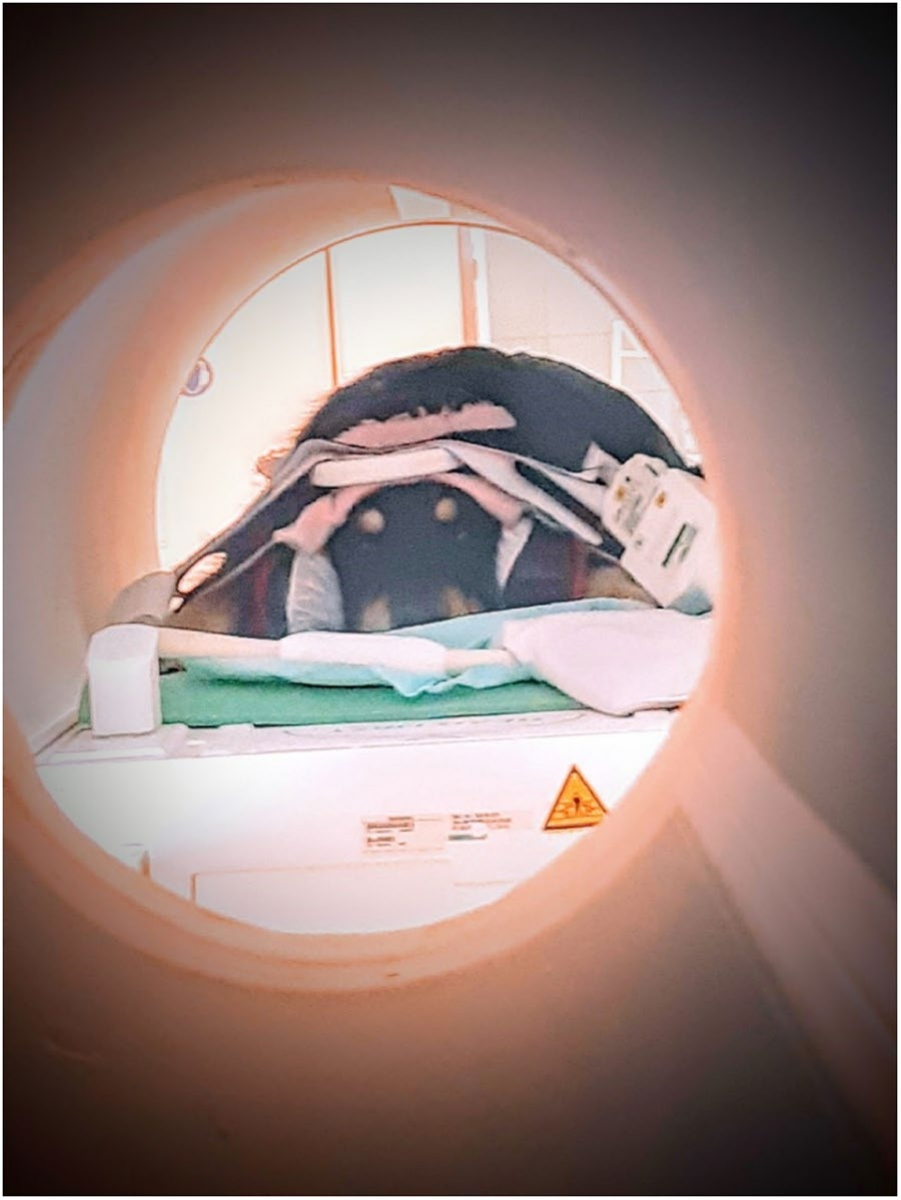
The scanning setup. The strap over the head was used to fixate the circular coil on the top of the dog’s head, it does not restrain the dog.

The experiment consisted of a 2-minute-long pretraining, to familiarize the dogs with the semi-continuous scanning procedure, and two 6-minute-long data collection runs. To provide sound protection, the dogs were wearing ear muffs. During scanning, dogs were lying with their eyes open, without presentation of a fixation cross, with their handler being visible, but avoiding eye contact with the subject. Figure 1 shows the dog’s in scanner position. The strap over the head was used to fixate the circular coil on the top of the dog’s head, not to restrain dog motion. Motion threshold for successful runs was set to a maximum of 2 mm (for each translation direction) and 2 degrees (for each rotation direction) during the whole run. During scanning, there was no eye contact between dog and owner, the owner was looking at the side of the scanner. Based on our experience, the dogs are usually not looking at the owner in this situation, the purpose of the presence of the human is to ensure that the dogs are comfortable and relaxed in this situation by providing dogs with a secure base. This relaxed state is essential to collect continous, 6 minutes long fMRI data from unrestrained dogs without repositioning under this motion treshold. Our extended training is based on the premise that the dogs are not being rewarded during scanning. This is crucial in our case, as expectation of immediate reward (and such an expectation would need to be sustained for over 6 minutes, as the dogs still keep performing the task) would result in increased salivation and swallowing, which would cause larger movements than our motion threshold. The dogs are conditioned to a specific ‘release procedure’ (the handler leaning into the bore of the scanner, unstrapping the coil from the dog’s head), which starts only after the scanning has stopped, so based on their training & scanning experience, the dogs do not expect immediate reward in this context.

### Ethics statement

All procedures were approved by the Ethical Committee of Eötvös Loránd University (KA-1719/PEI/001/1490-4/2015) and by the Government Office of Pest County Directorate of Food Chain Safety and Animal Health (XIV-I-001/520-4/2012), and conducted in accordance with relevant guidelines and regulations.

### Image acquisition

Functional MRI acquisitions were performed on a Siemens Prisma 3 T scanner (Siemens Healthcare, Erlangen, Germany) using a Gradient Echo Echo Planar Imaging (GRE-EPI) sequence with TR = 2640 ms including a 500-ms delay at the end of each volume, and TE = 30 ms. We decided to introduce the delay for welfare reasons. During piloting we realized that our dogs seem to find continuous scanning less comfortable than protocols with short gaps between subsequent scans. The 500 ms delay was selected empirically during piloting to include the shortest gaps that the dogs are still comfortable with. The protocol had an in-plane Field-of-View 128 mm × 128 mm, using 2 mm in-plane resolution and 2 mm slice thickness, measuring 31 slices with an inter-slice gap of 0.5 mm, using an excitation flip angle of 86°. Phase-encoding direction was set to left-right. A single loop coil (d = 11 cm) was used for signal detection, fixed onto the head of the dog and to the table of the scanner. In each run, 139 volumes were acquired, with the first 5 of them being discarded before processing, resulting in a total functional scanning time of 367 seconds/run. A T1-weighted anatomical scan was carried out separately as part of another study on each awake dog for spatial registration on a 3 T Philips Ingenia scanner (Philips Medical Systems, Best, The Netherlands), using a 3D Turbo Field Echo (TFE) sequence, with TR = 9.85 ms, TE = 4.6 ms, and an isotropic resolution of 1 mm.

### Image analysis

FMRI preprocessing included affine realignment (6 parameters, least square aproach) and reslicing of the images of the individual runs in SPM12 (http://www.fil.ion.ucl.ac.uk/spm/), followed by manual coregistration of the mean image to the individuals’ own structural T1 image in Amira 6.0 (Thermo Fisher Scientific). The individual structural images were normalized and transformed (linear, non-rigid transformation) to a stereotaxic breed-averaged, T2 weighted template brain^15^ with Amira. The resliced images were then coregistered and normalized to this transformed mean functional image via SPM’s standard nonlinear warping function with 16 iterations and smoothed with an FWHM of 4 mm.

**Figure 2:**
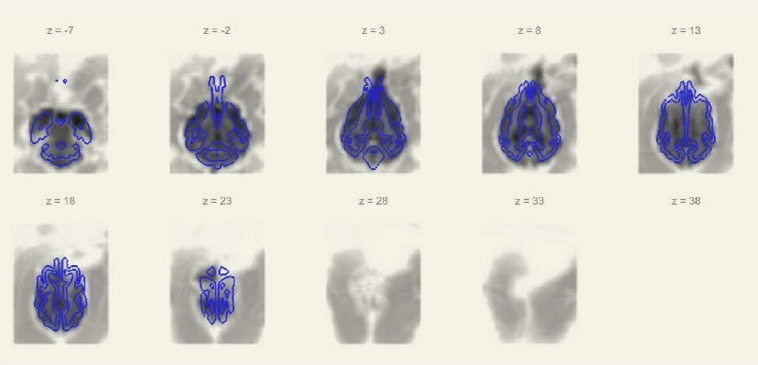
Sample of the coregistration of the grey matter mask to the mean functional image. The standard grey matter mask from the Nitzsche atlas^15^ was applied to the normalized images.

We applied band-pass filtering with a 0.01 and 0.1 Hz cutoff and linear detrending in CONN^16^. A single run had been censored due to exceeding motion threshold, after 127 scans (out of 134). As mean (scan-to-scan) motion was 0.035 ± 0.016 mm (*mean* ± *SD*), censoring of fMRI volumes during runs due to excessive motion, as applied by e.g.^17^ was deemed inappropriate, because only 3 volumes (each in separate runs) out of the 5975 collected images during the study (0.05%) would have been affected by such a treshold. We decided against censoring via removing volumes midrun from our functional datasets, because censoring in combination with frequency filtering can introduce additional artefacts^18^ and excessive motion was very infrequent (scan-to-scan displacement larger than 0.2 mm occurred in only 5% of the scans). We applied published, averaged, segmented white matter (WM) and cerebrospinal fluid (CSF) dog MRI brain masks^15^ in our analysis as nuisance regressors to filter out non-neural signal fluctuations. These standard masks were applied to our normalized images. Figure 2 shows a sample of the coregistration of the grey matter mask to a functional image.

### Independent component analysis

**Figure 3:**
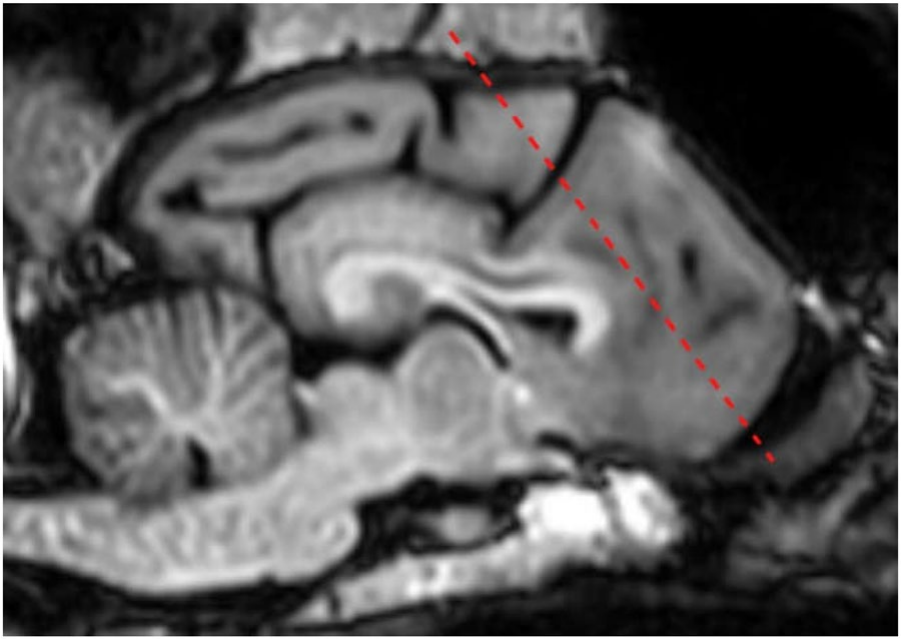
Guideline to flag clusters with a high-risk of susceptibility. We considered the dashed red line as the boundary corresponding to the largest extent of the susceptibility artefact, clusters with a center (investigated with multiple thresholds) anterior to this line would have been considered as susceptibility artefact.

Independent Component Analysis (ICA) is a model-free, data driven method. Spatial ICA seeks components that are systematically non-overlapping, temporally coherent and maximally independent in space, without constraining the shape of the temporal response. We used the CONN software^16^ to carry out group level spatial ICA. CONN is a Matlab-based cross-platform software for the computation, display, and analysis of functional connectivity in fMRI. ICA identifies a number of networks of highly functionally-connected areas. CONN’s implementation uses Calhoun’s group-level ICA approach^19^, with variance normalization pre-condititioning, subject concatenation of BOLD signal data along temporal dimension, group-level dimensionality reduction (to the target number of dimensions/components), fastICA for estimation of independent spatial components, and GICA1 backprojection for individual subject-level spatial map estimation. We included the WM mask, CSF mask and the realignment parameters to estimate physiologic noise (e.g. cardiac and respiratory cycles) and included them as nuisance regressors to filter out non-neural signal fluctuations. We applied a whole-brain mask to restrict the analysis space. Before the analysis, we also inspected QA plots regarding registration of the normalised functional images and the outline of the grey matter ROI. Anatomical labelling was carried out based on relevant anatomical brain atlases^15,20–22^. During evaluation of the components, we relied on the guidelines published in Griffanti *et al*.^23^. We inspected the components with multiple thresholds, with different planes, while looking at both positive and negative clusters. During evaluating the components, we took into account the location of the susceptibility artefact and took a conservative approach to avoid classifying components containing clusters predominantly from the affected area as neural signal. As currently there is little information regarding the location and extent of susceptibility artefact in case of different dog breeds’ functional brain scans, we created a guideline after going through and summarizing our raw images, looking for the regions which were affected by distortion/signal loss in the individual runs. Figure 3 shows the guideline we utilized during our evaluation, to flag clusters which are likely to be the result of susceptibility. While increasing model order increases the functional neuroanatomical precision, it reduces the repeatability of the ICA decomposition^24^. As model orders of 10–20 were reported as most suitable to detect large functional network clusters^24^, we run gICA with model orders of 10, 15 and 20.

### Assessing reproducibility of independent components

To evaluate the reproducibility of the resulting ICA components, we calculated the Dice similarity coefficient. The Dice similarity coefficient (DSC) is used to evaluate reliability or reproducibility of MRI volumes, providing a reproducibility validation metric via calculating a spatial overlap index. The value of a DSC ranges from 0, indicating no spatial overlap between two sets of binary segmentation results, to 1, indicating complete overlap^25^. In case of evaluating consistency of resting-state networks from ICA, a DSC value ≥0.3 is considered as good spatial overlap consistency in studies explicitly designed to investigate reproducibility^26^.

To get an estimate of spatial overlap consistency of our data, we split it into two parts (first and second run of the subjects), then we run two additional, separate subICAs and calculated the spatial overlap between these sets and the original ICA. First, to match the subICA components to the originals, we compared them to the original ICA components, and labelled them based on the followings: the ICA component having the highest DSC value for the original network component X was labelled X. If a component had the highest DSC value for multiple networks, the label with the highest DSC value was selected. If the highest DSC value for a network was below 0.3, it was labelled with a new letter.

## Results

To evaluate the quality of the collected data regarding head motion, we calculated mean motion from the realigment parameters. In our sample mean motion was 0.035 mm ± 0.016 mm (*mean* ± *SD*, n = 22), which is comparable to the reported mean motion values in human samples^27^. This confirms the effectiveness of our training procedure and shows the feasibility of carrying out resting-state measurements with awake unrestrained dogs.

### Independent component analysis

**Figure 4:**
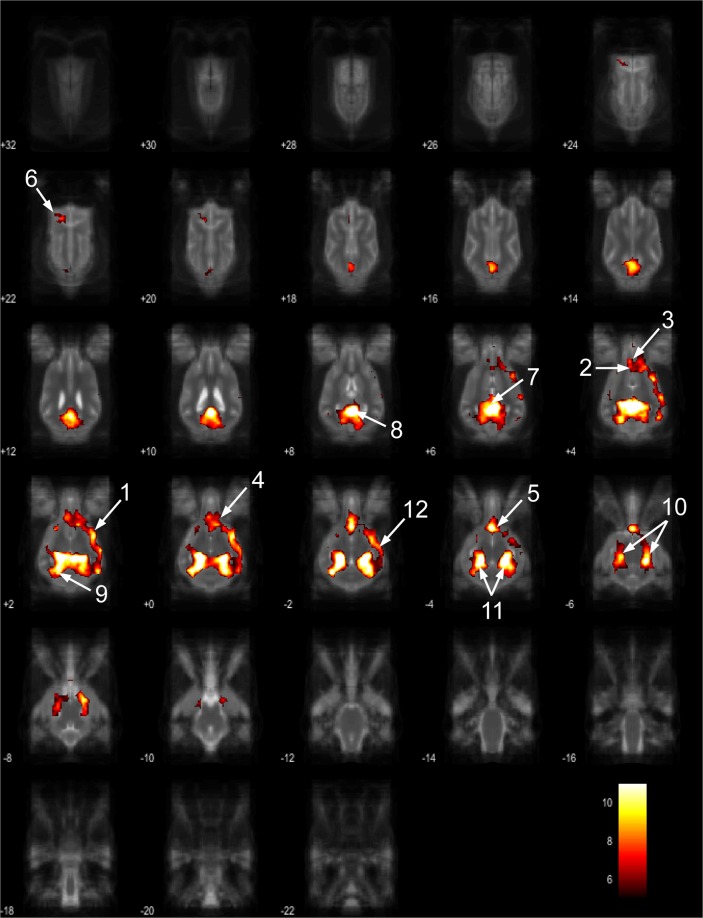
Resting-state network component A from the 15 component gICA. We report the results with a voxel-wise FDR corrected p < 0.001 and cluster treshold of FDR corrected p <0.005. gICA components are presented as thresholded T-maps, corrected for multiple comparisons, overlaid on the Nitzsche atlas^15^. 1. rostral composit gyrus, 2. cingulate gyrus, 3. straight gyrus, 4. Subcallosal area, 5. diagonal gyrus, 6. Left premotor area, 7. Medial cingulate gyrus, 8. Bilateral caudal regions of the cingulate gyrus, 9. splenial gyrus, 10. bilateral hippocampus, 11. bilateral parahippocampal gyrus, 12. caudal composit gyrus.

**Figure 5:**
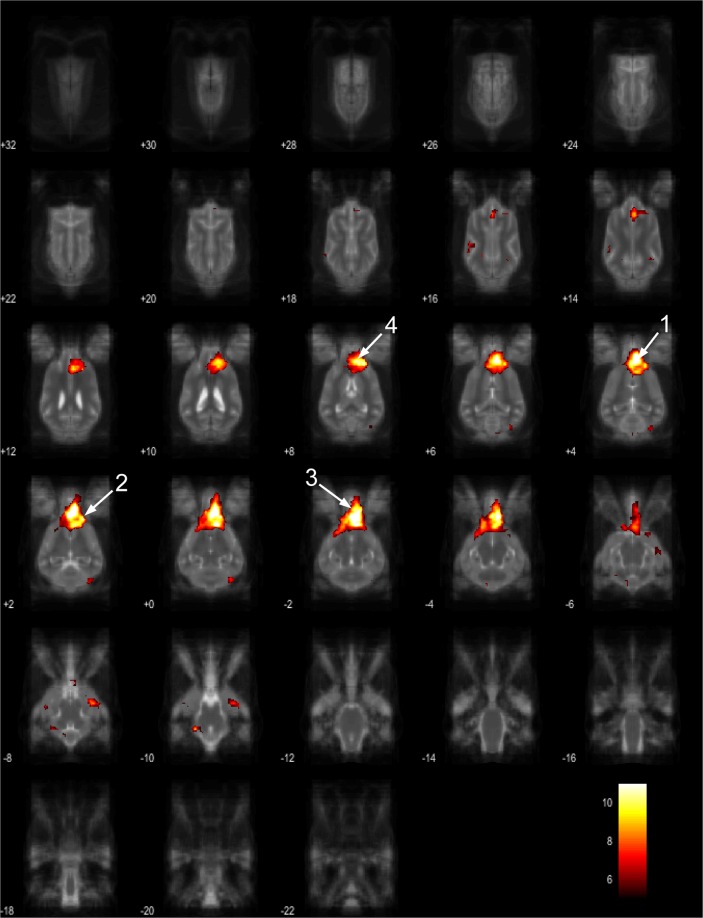
Resting-state network component B from the 15 component gICA. We report the results with a voxel-wise FDR corrected p < 0.001 and cluster treshold of FDR corrected p < 0.005. gICA components are presented as thresholded T-maps, corrected for multiple comparisons, overlaid on the Nitzsche atlas^15^. 1. genual gyrus, 2. prorean gyrus, 3. straight gyrus, 4. dorsal anterior cingulate cortex.

**Figure 6:**
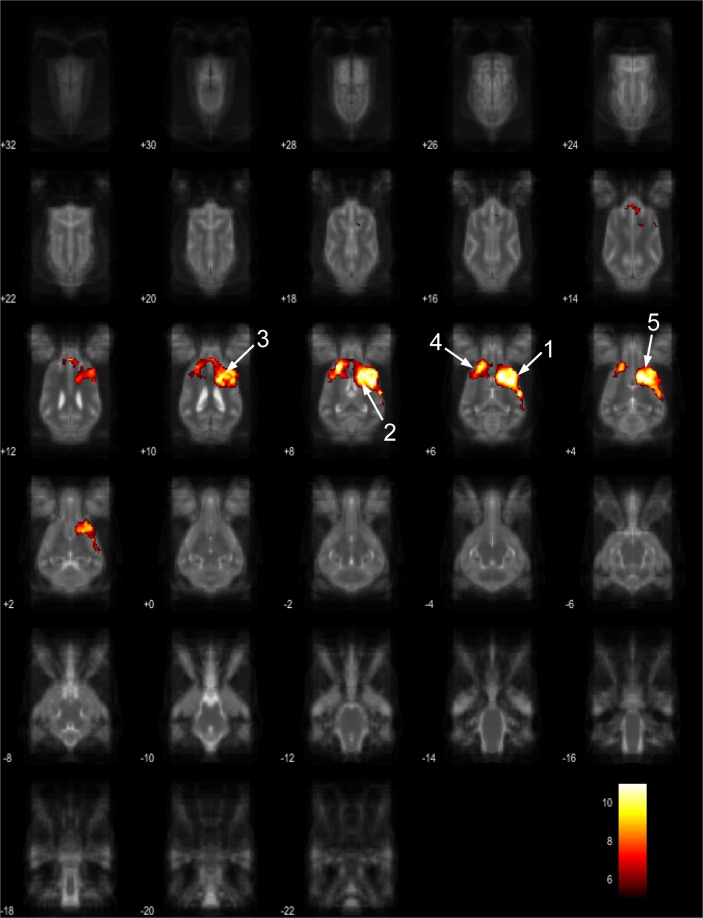
Resting-state network component C from the 15 component gICA. We report the results with a voxel-wise FDR corrected p < 0.001 and cluster treshold of FDR corrected p < 0.005. gICA components are presented as thresholded T-maps, corrected for multiple comparisons, overlaid on the Nitzsche atlas^15^. 1. right rostral composit gyrus, 2. right caudate nucleus, 3. right rostral suprasylvian gyrus, 4. left rostral composit gyrus, 5. prorean gyrus.

**Figure 7:**
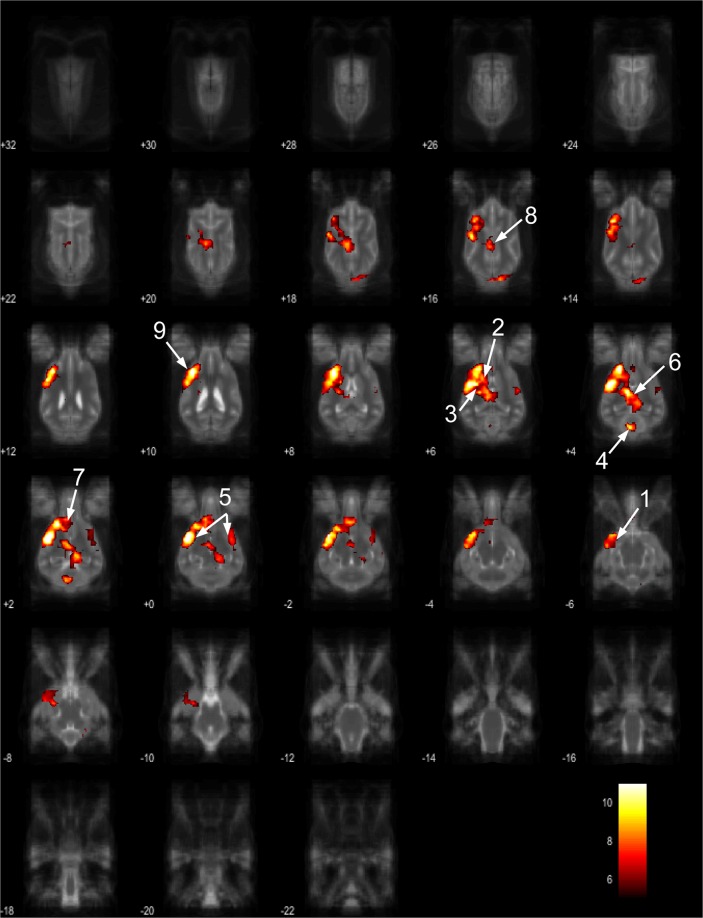
Resting-state network component D from the 15 component gICA. We report the results with a voxel-wise FDR corrected p < 0.001 and cluster treshold of FDR corrected p < 0.005. gICA components are presented as thresholded T-maps, corrected for multiple comparisons, overlaid on the Nitzsche atlas^15^. 1.left amygdala, 2. left caudate nucleus, 3. striatum, 4. cerebellum, 5. bilateral insular cortex, 6. thalamus, 7. medial prefrontal cortex, 8. mid-cingulate gyrus, 9. left rostral composit gyrus.

**Figure 8:**
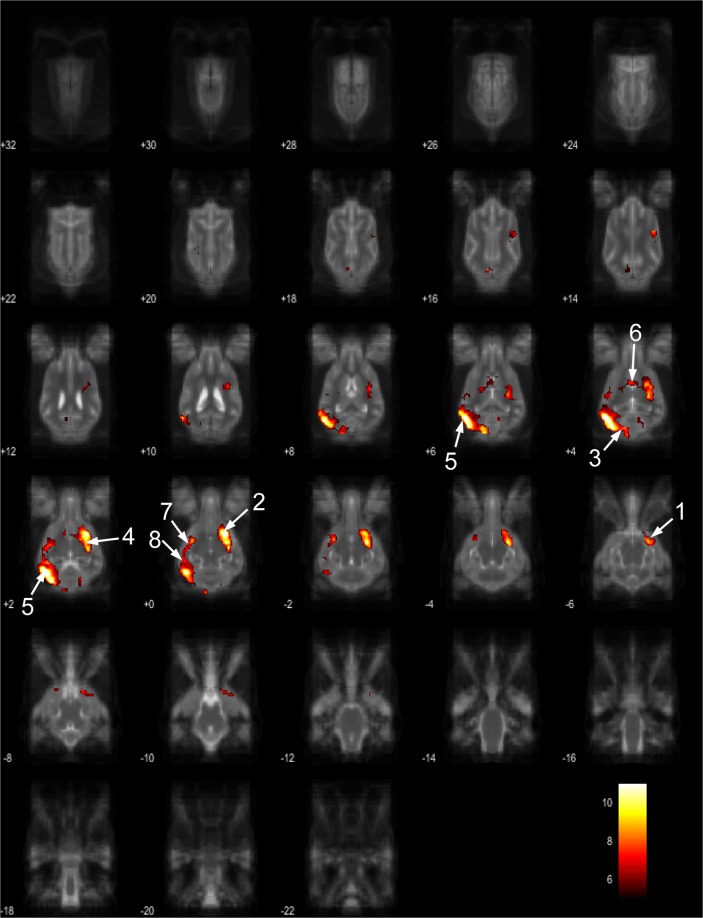
Resting-state network component E from the 15 component gICA. We report the results with a voxel-wise FDR corrected p < 0.001 and cluster treshold of FDR corrected p < 0.005. gICA components are presented as thresholded T-maps, corrected for multiple comparisons, overlaid on the Nitzsche atlas^15^. 1. right amygdala, 2. Striatum, 3. Cerebellum, 4. right insular cortex, 5. left visual cortex, 6. septal nuclei, 7. left sylvian gyrus, 8. left ectosylvian gyrus.

**Figure 9:**
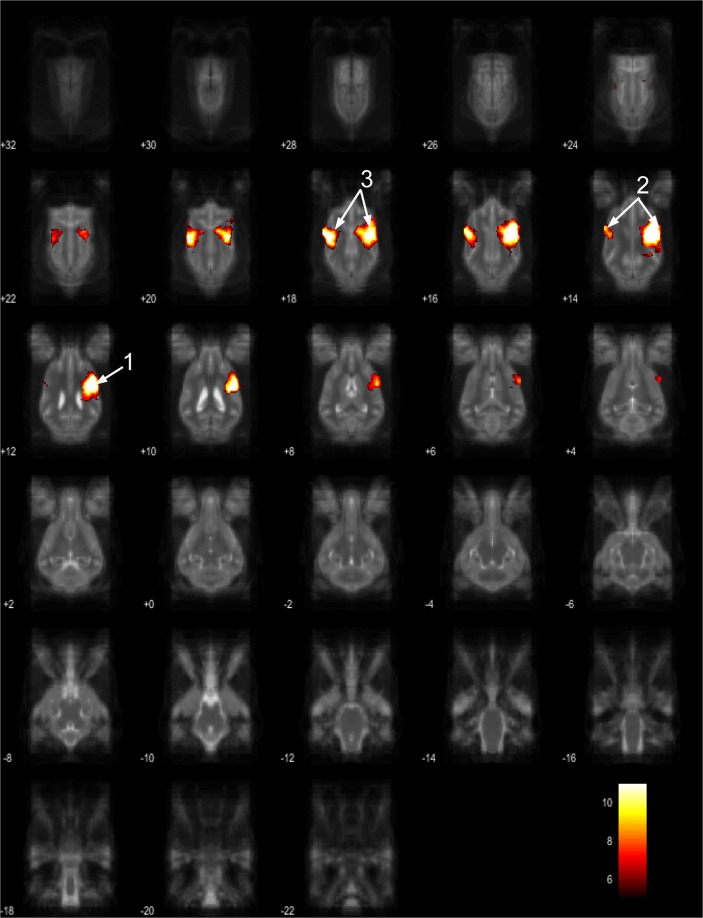
Resting-state network component F from the 15 component gICA. We report the results with a voxel-wise FDR corrected p < 0.001 and cluster treshold of FDR corrected p < 0.005. gICA components are presented as thresholded T-maps, corrected for multiple comparisons, overlaid on the Nitzsche atlas^15^. 1. sensorimotoric cortex, 2. bilateral rostral ectosylvian gyrus, 3. rostral suprasylvian gyrus.

**Figure 10:**
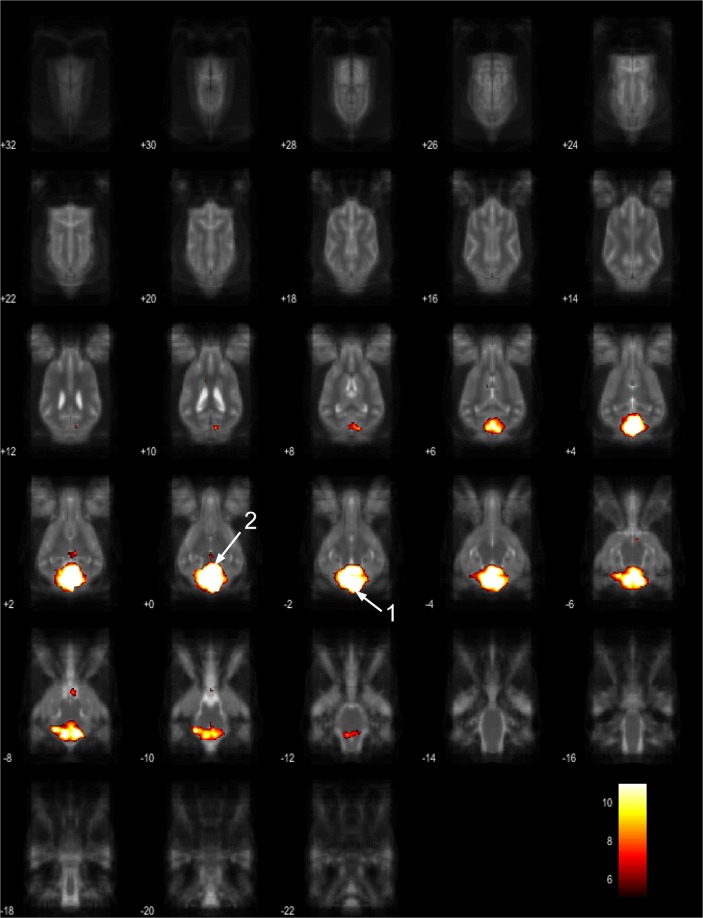
Resting-state network component G from the 15 component gICA. We report the results with a voxel-wise FDR corrected p < 0.001 and cluster treshold of FDR corrected p < 0.005. gICA components are presented as thresholded T-maps, corrected for multiple comparisons, overlaid on the Nitzsche atlas^15^. 1. cerebellum, 2. corpora quadrigemina.

**Figure 11:**
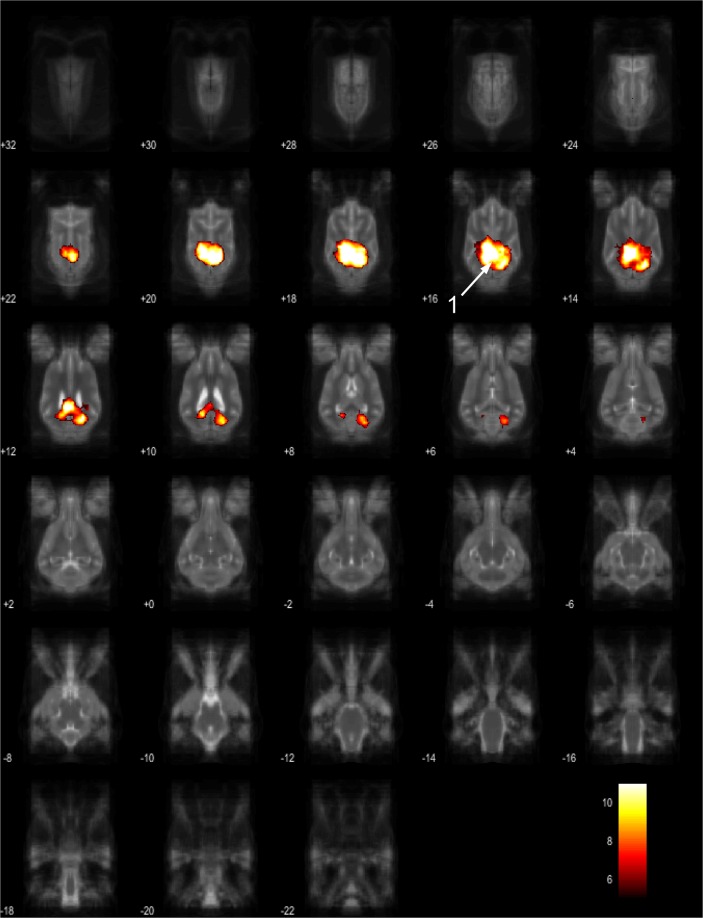
Resting-state network component H from the 15 component gICA. We report the results with a voxel-wise FDR corrected p < 0.001 and cluster treshold of FDR corrected p < 0.005. gICA components are presented as thresholded T-maps, corrected for multiple comparisons, overlaid on the Nitzsche atlas^15^. 1. mid-cingulate cortex.

**Figure 12:**
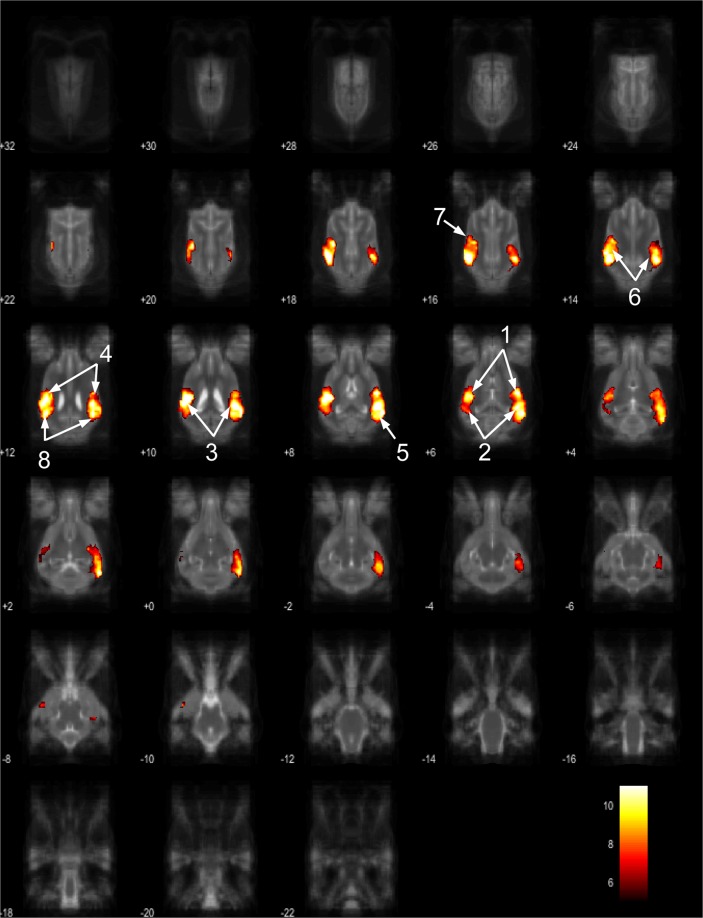
Resting-state network component I from the 15 component gICA. We report the results with a voxel-wise FDR corrected p < 0.001 and cluster treshold of FDR corrected p < 0.005. gICA components are presented as thresholded T-maps, corrected for multiple comparisons, overlaid on the Nitzsche atlas^15^. 1. rostral sylvian gyrus, 2. caudal sylvian gyrus, 3. middle ectosylvian gyrus, 4. rostral ectosylvian gyrus, 5. caudal ectosylvian gyrus, 6 middle suprasylvian gyrus, 7. rostral suprasylvian gyrus, 8. caudal suprasylvian gyrus.

**Figure 13:**
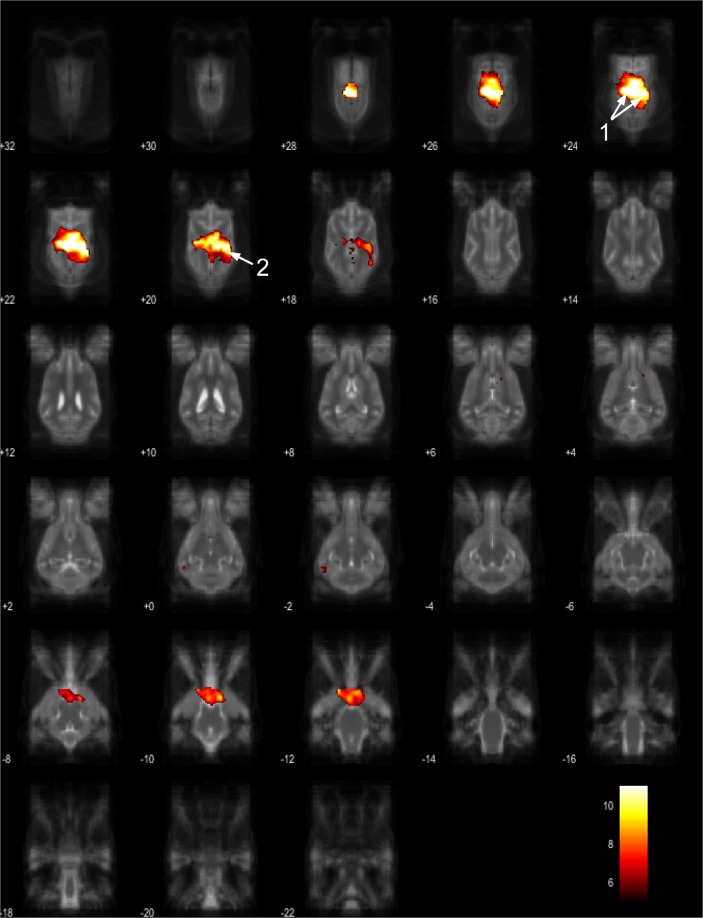
Resting-state network component J from the 15 component gICA. We report the results with a voxel-wise FDR corrected p < 0.001 and cluster treshold of FDR corrected p < 0.005. gICA components are presented as thresholded T-maps, corrected for multiple comparisons, overlaid on the Nitzsche atlas^15^. 1. bilateral marginal gyrus, 2. ectomarginal gyrus.

**Figure 14:**
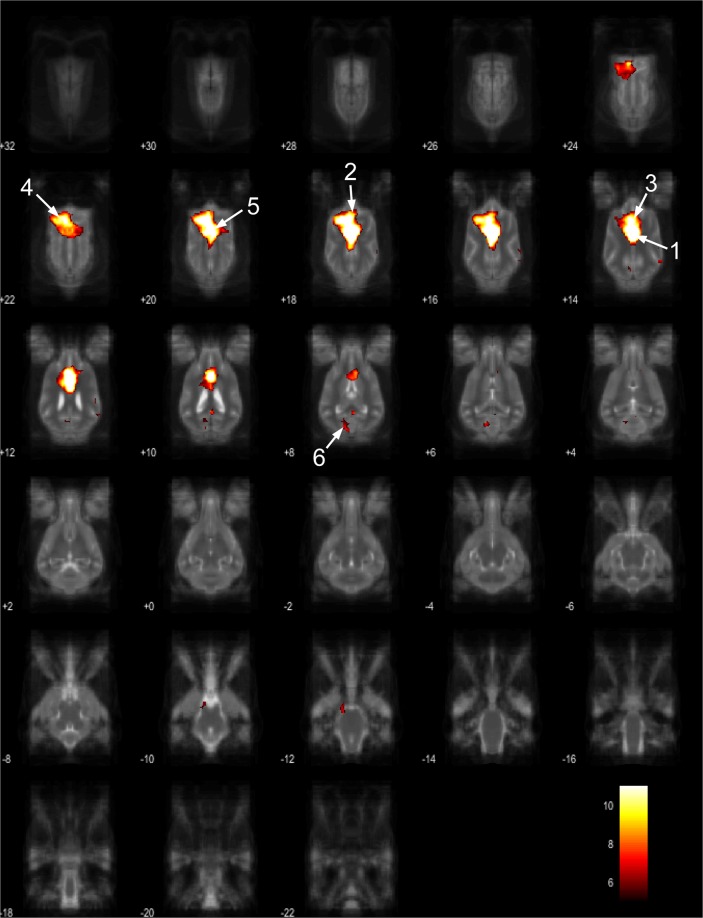
Resting-state network component K from the 15 component gICA. We report the results with a voxel-wise FDR corrected p < 0.001 and cluster treshold of FDR corrected p < 0.005. gICA components are presented as thresholded T-maps, corrected for multiple comparisons, overlaid on the Nitzsche atlas^15^. 1. mid-cingulate gyrus, 2. frontal gyrus, 3. gyrus genualis, 4. precruciate gyri, 5. postcruciate gyri, 6. splenial gyrus.

**Figure 15:**
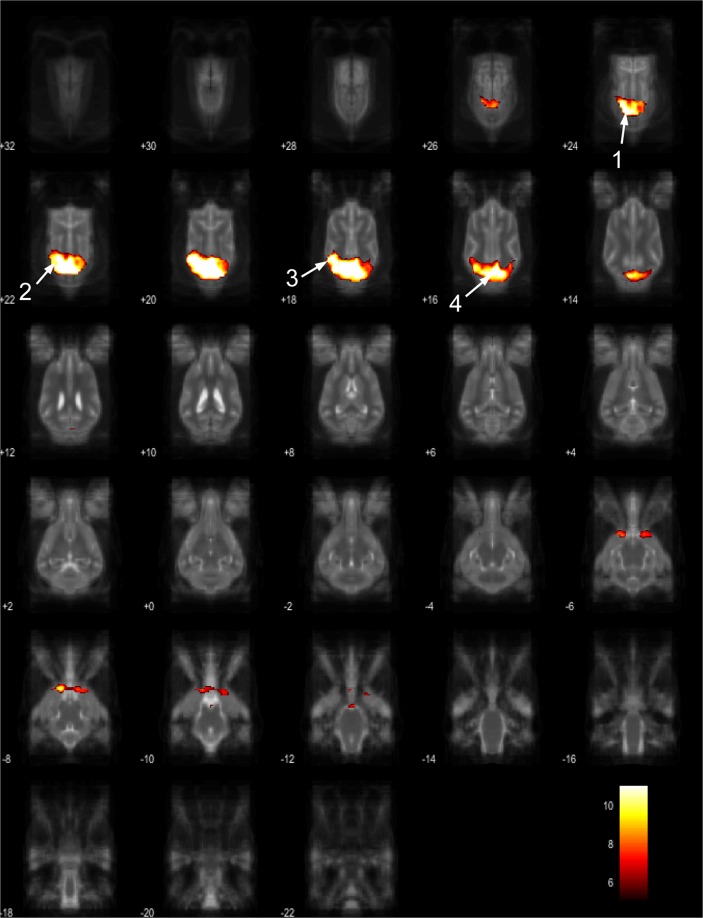
Resting-state network component L from the 15 component gICA. We report the results with a voxel-wise FDR corrected p < 0.001 and cluster treshold of FDR corrected p < 0.005. gICA components are presented as thresholded T-maps, corrected for multiple comparisons, overlaid on the Nitzsche atlas^15^. 1. marginal gyrus, 2. ectomarginal gyrus, 3. suprasylvian gyrus, 4. occipital gyrus.

**Figure 16:**
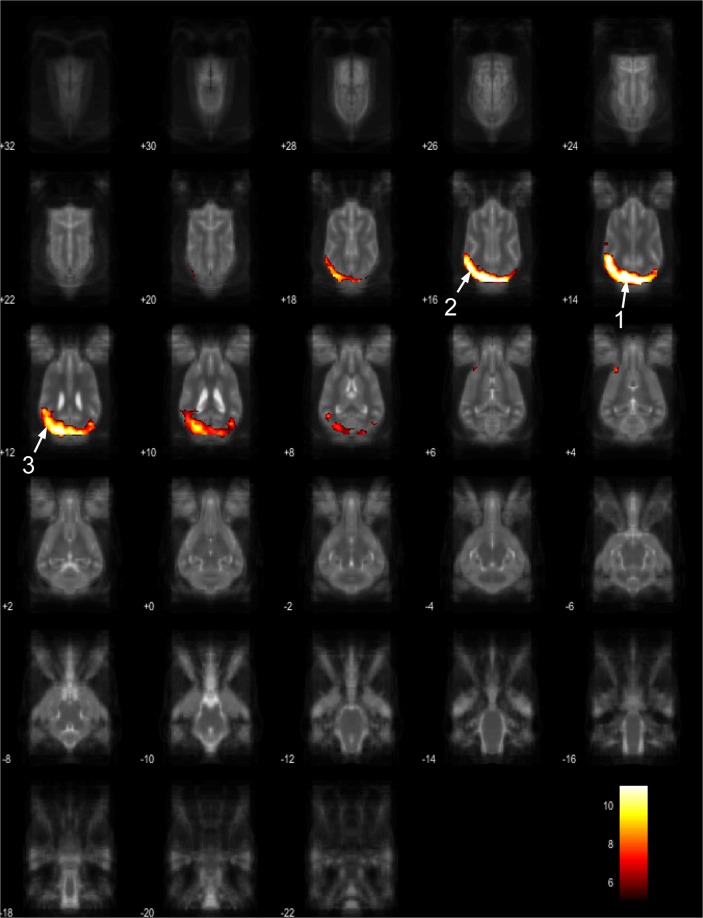
Resting-state network component M from the 15 component gICA. We report the results with a voxel-wise FDR corrected p < 0.001 and cluster treshold of FDR corrected p < 0.005. gICA components are presented as thresholded T-maps, corrected for multiple comparisons, overlaid on the Nitzsche atlas^15^. 1. occipital gyrus, 2. gyrus ectomarginalis, 3. caudal suprasylvian gyrus.

**Figure 17:**
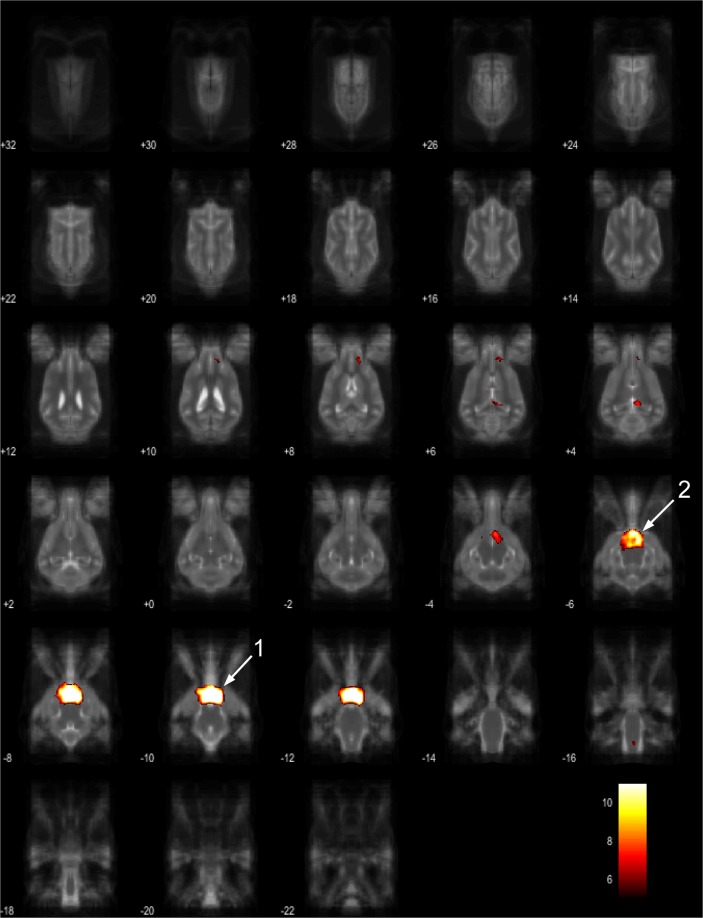
Resting-state network component N from the 15 component gICA. We report the results with a voxel-wise FDR corrected p < 0.001 and cluster treshold of FDR corrected p < 0.005. gICA components are presented as thresholded T-maps, corrected for multiple comparisons, overlaid on the Nitzsche atlas^15^. 1. sinus cavernosus, 2. Willis’ circle.

**Figure 18:**
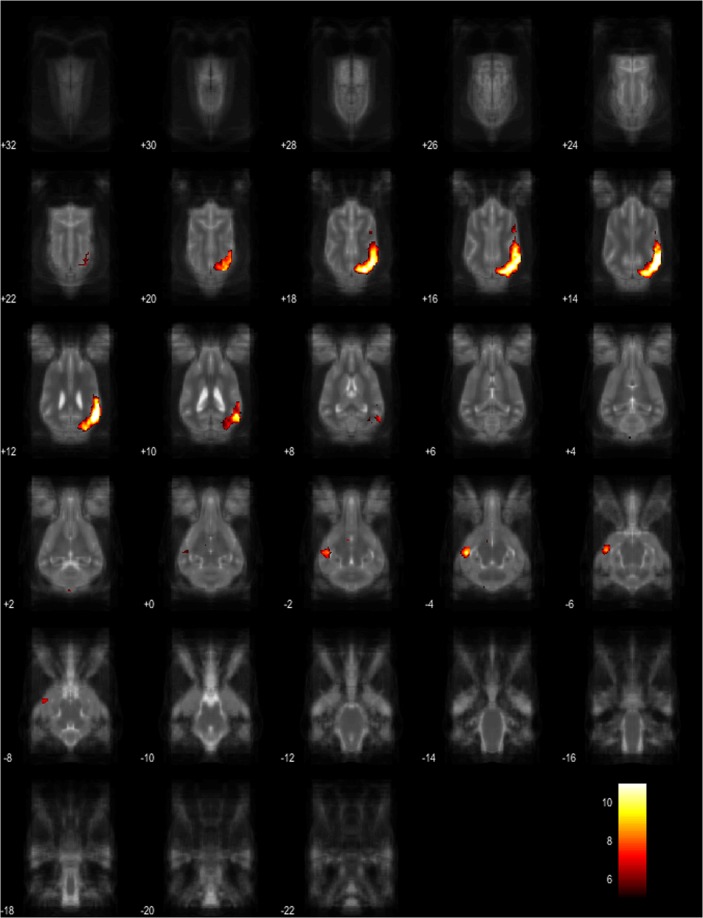
Resting-state network component O from the 15 component gICA. We report the results with a voxel-wise FDR corrected p < 0.001 and cluster treshold of FDR corrected p < 0.005. gICA components are presented as thresholded T-maps, corrected for multiple comparisons, overlaid on the Nitzsche atlas^15^.

The 15 component group level independent component analysis (gICA) contained thirteen components which showed characteristics of containing signals sources from primary of neural origin (Figs 4–16). We report regions surviving controlling for multiple comparisons with a voxel-wise threshold of p-FDR corrected <0.001 and a cluster threshold of p-FDR corrected <0.005. Component A (Fig. 4) covered parts of the rostral composit gyrus, rostral regions of the cingulate gyrus and the straight gyrus, the subcallosal area and diagonal gyrus; the left premotor area; medial and bilateral caudal regions of the cingulate gyrus and splenial gyrus, bilateral regions of the hippocampus and parahippocampal gyrus and the caudal composit gyrus. Component B (Fig. 5) included anterior brain regions in the prefrontal area, namely the genual gyrus, prorean gyrus, straight gyrus and dorsal anterior cingulate cortex. This component was located dorsally from the frontal hubs of Component A. Component C (Fig. 6) was a right lateralized frontal network, including secondary somatosensory cortices, such as the right rostral composit gyrus, the right caudate nucleus and the right rostral suprasylvian gyrus. While this network was less extensive on the left side, it showed clear indications of bilaterality, covering parts of the frontal lobe, namely the left rostral composit gyrus and the prorean gyrus. We found two components which were in part symmetrical, one of them left while the other right lateralized. Component D (Fig. 7) was left lateralized, including the left amygdala, left caudate nucleus, striatum, cerebellum, bilateral insular cortex, thalamus, fronto-parietal regions, the medial prefrontal cortex, and the mid cingulate gyrus. Component E (Fig. 8) covered the right amygdala, the striatum, the cerebellum, the right insular cortex, parts of the left visual corex, the septal nuclei, the left sylvian and ectosylvian gyrus. Component F (Fig. 9) covered regions at the junction of the frontal, temporal and parietal lobes, including frontal regions of the sensorimotoric cortex, the bilateral rostral ectosylvian gyrus and rostral suprasylvian gyrus. Component G (Fig. 10) covered the cerebellum and the mesencephalon (corpora quadrigemina). This component is analogue to cerebellar networks previously reported in human^28^ and non-human animal studies^6,8^. Component H (Fig. 11) consisted of the mid cingulate cortex, an integrative part of the external limbic circle, related to affective processes and memory. Component I (Fig. 12) covered the bilateral auditory cortices, namely the rostral and caudal part of the sylvian gyrus; middle, rostral and caudal parts of the ectosylvian gyrus; and the middle, rostral and caudal regions of the suprasylvian gyrus. Component J (Fig. 13) included the primer and associative sensory cortical areas, namely the bilateral marginal gyrus and the ectomarginal gyrus. Component K (Fig. 14) included the mid cingulate gyrus, frontal gyrus, genual gyrus, the pre- and postcruciate gyri, the splenial gyrus, the posterior cingulate gyrus and the parahippocampal gyrus. These regions correspond to the primer sensorimotoric, premotoric and supplementer motoric regions of the dog brain. Component L (Fig. 15) covered primary and secondary visual areas, sensory and visual-sensorimotor cortices, namely the marginal gyrus, the ectomarginal gyrus, the suprasylvian gyrus and occipital gyrus. Component M (Fig. 16) included primary visual areas, such as the occipital gyrus, ectomarginal gyrus and caudal suprasylvian gyrus. Component L & M both are networks consisting of regions involved in visual processing. Component N (Fig. 17) was classified as a noise component corresponding to large vessels. It covered the sinus cavernosus and the Willis’ circle, large veins and arteries located at the ventral part of the brain. Component O (Fig. 18) showed characteristics consistent with a motion artefact (a ring around the edge of the brain). We classified component O as motion artefact because it contained a ring around the edge of the brain (which was more visible when both the positive and negative clusters are displayed as they are complementary) and the clusters did not follow known anatomical boundaries. In contrast, component M did not show these characteristics, it also contained bilateral frontal clusters which were not ring-like (slices 4–8) and component M showed a pattern which was overlapping with brain regions known to process stimuli from the visual modality^29–31^.

**Figure 19:**
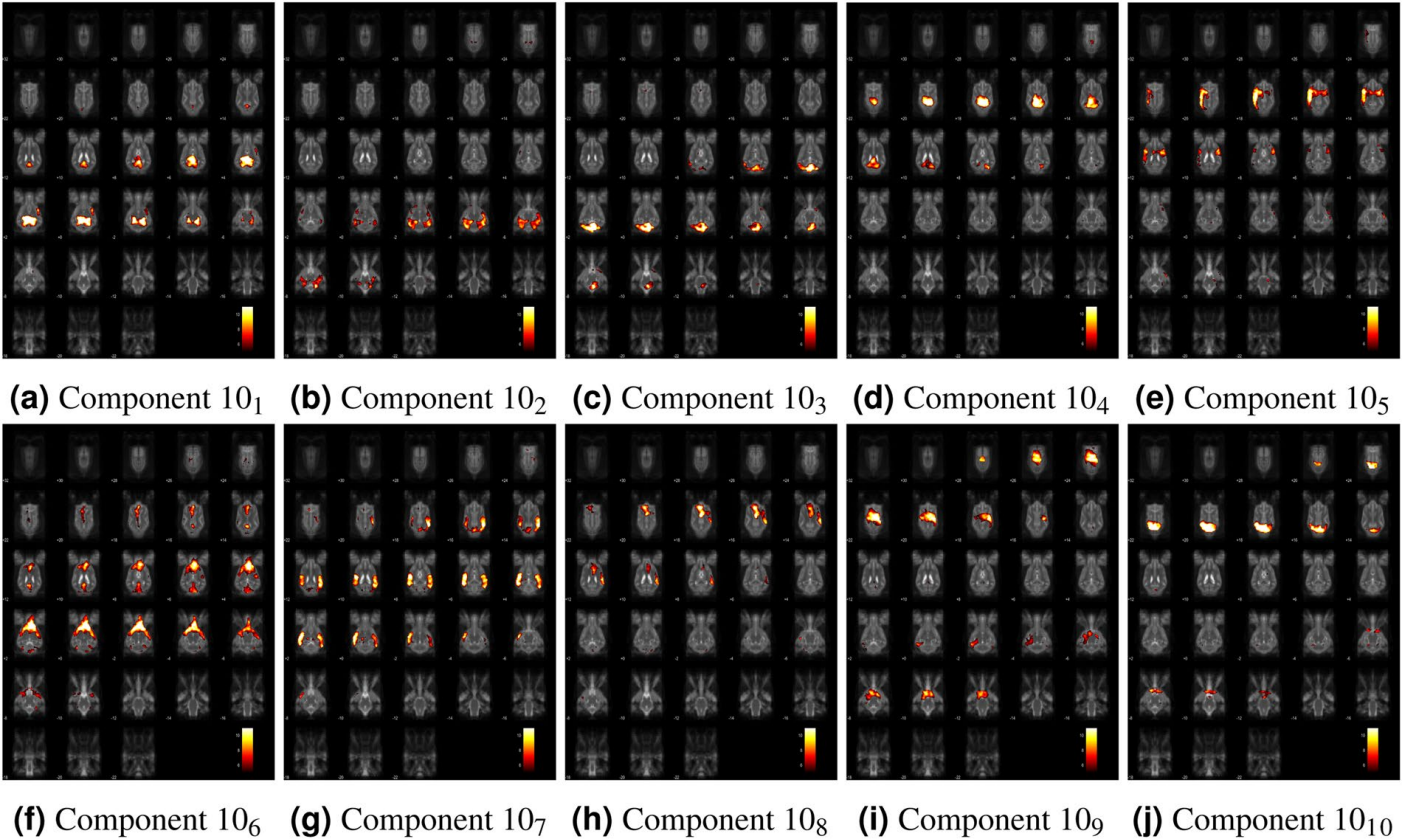
Components from the 10 component gICA. Thresholded T-maps, corrected for multiple comparisons, overlaid on the Nitzsche atlas^15^.

Compared to the 15 component gICA, the 10 item gICA contained only one miscancellous/noise component, while multiple components showed signs of mixed signal sources merged together, rendering this modell less conlcusive (Table 1, Fig. 19). At model order 20, the cerebellar and medial visual components were both split in two, while the other components remained similar.

**Table 1:** Spatial topography of ICA models at model orders of 15 and 10, listing the most characteristic brain regions.

Component	Involved brain areas	Similar components in gICA_10_
A	Rostal and caudal parts of the cingulate gyrus	1,2,6
B	Prefrontal area	—
C	Frontal lobe	5
D	Striatum (left lateralized)	—
E	Striatum (rigth lateralized)	—
F	Frontal regions of the sensorimotoric cortex	5
G	Cerebellum	3
H	External limbic circle (mid cingulate cortex)	—
I	Bilateral auditory cortices	7
J	Primer and associative sensory cortical areas	9
K	Primer sensorimotoric, premotoric and supplementer motoric regions	8
L	Sensory and visual-sensorimotor cortices	7,10
M	Occipital lobe	4
N	Artefact (large vessels)	—
O	Artefact (motion)	—

Capital letters refer to components from gICA_15_, while the numbers refer to components from the gICA_10_. Similarity is based on the involved brain regions and visual characteristics.

### Assessing reproducibility of independent components

#### Labelling subset components

In subset 1 (Table 2), no component was labelled as O, and in subset 2 (Table 3), no components were labelled as E, M (this component fused with L, the other visual component), and O. These nonmatching components usually were some type of noisy combinations of the original networks (e.g. component Y in subset 2 contained parts of component A and F). The only original component which had no match in either of the subsets was component O, which we originally classified as a motion artefact.

**Table 2:** Dice similarity coefficients used for labelling the components of subset 1.

Labelled component in subset 1	A	B	C	D	E	F	G	H	I	J	K	L	M	N	X
**Network**															
A	**0.47**	0.22	0.09	0	0.01	0.02	0.12	0.01	0.12	0.01	0.05	0.05	0.03	0.07	0.13
B	0	**0.47**	0.16	0	0.03	0	0.04	0.01	0.02	0	0.06	0	0.01	0.08	0.01
C	0	0.07	**0.44**	0.09	0	0.21	0	0	0.09	0	0.04	0	0.01	0.01	0
D	0.1	0.19	0.17	**0.35**	0.01	0.03	0.05	0.08	0.08	0.04	0.03	0.02	0.06	0	0.02
E	0.03	0.07	0.14	0	**0.38**	0.07	0.07	0	0.1	0	0.02	0.02	0.02	0.02	0.07
F	0	0.02	0.01	0.23	0	**0.71**	0	0	0.11	0.16	0.09	0	0.02	0	0.03
G	0.15	0	0	0	0.05	0	**0.76**	0	0	0	0	0	0.01	0.03	0.03
H	0.18	0.03	0	0.01	0	0	0	**0.42**	0.02	0.07	0.02	0.29	0	0	0
I	0	0.01	0.01	0.1	0.06	0.05	0	0	**0.74**	0.03	0.01	0.03	0.13	0	0.04
J	0	0.01	0.01	0.04	0	0.08	0	0.09	0.03	**0.76**	0.07	0.24	0	0.26	0.06
K	0.06	0.02	0.05	0.15	0	0.07	0.01	0.08	0.02	0.15	**0.73**	0.01	0	0.01	0
L	0.05	0	0	0	0.01	0.01	0	0.05	0.02	0.04	0.02	**0.77**	**0.32**	0.09	0.22
M	0.06	0	0.01	0.03	0.04	0	0	0	0.06	0	0.02	0.1	**0.74**	0	0.01
N	0.04	0.05	0	0	0.01	0.01	0	0	0	0.12	0.01	0	0	**0.72**	0.01
O	0	0	0	0	0	0.06	0.01	0	0.26	0.04	0.05	0.22	0.05	0	0

Dice similarity coefficients over 0.3 are highlighted in bold.

**Table 3:** Dice similarity coefficients used for labelling the components of subset 2.

Labelled component in subset 2	A	B	C	D	F	G	H	I	J	K	L	N	Y	Z	V
**Network**															
A	**0.41**	0.09	0.1	0.05	0.01	0.05	0	0.16	0	0.03	0.04	0.09	0.27	0.18	0.11
B	0	**0.34**	**0.31**	0.01	0.06	0.03	0.02	0.11	0	0.04	0	0.03	0	0.23	0.16
C	0	0.03	**0.52**	0.22	0	0	0	0.12	0	0.02	0	0	0.06	0	0.04
D	0.08	0.05	0.01	**0.5**	0.07	0.05	0.09	0.05	0.03	0.04	0.04	0.01	0.08	0.13	0.02
E	0.05	0	0.04	0.06	0.06	0.06	0.01	0.02	0	0.02	0.02	0.02	0.1	0.14	0.11
F	0	0	0.06	0.02	**0.32**	0	0.12	0	0.09	0.12	0	0	0.19	0	0.01
G	0.14	0	0	0.01	0	**0.77**	0	0.03	0	0	0	0.02	0.09	0.01	0.02
H	**0.35**	0	0	0.05	0	0	**0.56**	0	0.14	0.02	0.13	0	0.02	0	0.16
I	0	0	0.01	0.04	0.25	0	0.07	**0.37**	0.04	0.1	0.03	0	0.09	0.01	0.08
J	0.02	0	0.01	0.03	0.11	0	0.13	0	**0.73**	0.04	0.15	0.27	0.06	0	0.06
K	0.12	0	0.18	0.12	0.06	0.01	0.05	0	0.08	**0.55**	0	0.01	0.01	0	0.08
L	0.06	0	0	0.01	0.08	0	0.11	0	0.21	0	**0.81**	0.12	0	0.03	0.14
M	0.04	0	0	0.03	0.15	0	0	0	0	0	0.22	0	0	0	0.05
N	0.04	0.03	0.02	0.01	0	0	0	0.03	0	0	0.03	**0.85**	0	0.03	0.02
O	0.01	0	0	0	0.01	0.01	0.01	0.07	0.08	0.18	0.18	0	0.11	0	0.17

Dice similarity coefficients over 0.3 are highlighted in bold.

#### Reproducibility across runs

After this, we calculated the DSC value between the two, labelled subICA components (Table 4). Most of the RSNs (A, C, D, G, H, I, J, K, L) had good spatial overlap consistency (DSC > 0.25) except B (0.15) and F (0.16) which were also overlapping with some nonmatching components. The low DSC value of B is most likely a result of that in subset 2, two separate components showed a DSC value higher than 0.3. As our study was not designed to carry out this analysis (hence the low number of datapoints), this high consistency in spatial overlap between separate ICAs shows the robustness and reproducibility of the reported components.

**Table 4:** Dice similarity coefficients of the two ICAs, run separately for the two runs.

Subset1	A	B	C	D	F	G	H	I	J	K	L	N	Y	Z	V
**Subset2**															
A	**0.58**	0.02	0	0.06	0	0.1	0.03	0.05	0.01	0.01	0.03	0.03	**0.27**	0.13	0.08
B	0.14	0.15	0.12	0.12	0.12	0.08	0.15	0.07	0.08	0.06	0.05	0.04	0.08	0.17	0.11
C	0.04	0.08	**0.33**	0.19	0	0.01	0.01	0.12	0.02	0	0.01	0.02	0.05	0.04	0.07
D	0.03	0.01	0	**0.4**	**0.3**	0	0.1	0	0.03	0.08	0.04	0	0.01	0.01	0
E	0.04	0	0	0.03	0.11	0.06	0.02	0.02	0.01	0	0.03	0.03	0.02	0.04	0.08
F	0.02	0	0.17	0.04	0.16	0.01	0.04	0.01	0.04	0.14	0.04	0.03	0.17	0	0.01
G	0.15	0.01	0.02	0.04	0.01	**0.59**	0	0.02	0	0.01	0.03	0.03	0.1	0.04	0.03
H	0.15	0	0	0.08	0.01	0	**0.48**	0	0.07	0.05	0	0	0	0	0.07
I	0.03	0.02	0.02	0.06	0.17	0.02	0.06	**0.26**	0.05	0.11	0.06	0.05	0.12	0.04	0.08
J	0.02	0	0.01	0.03	0.17	0.01	0.1	0	**0.5**	0.1	0.05	**0.28**	0.1	0	0.02
K	0.14	0	0.17	0.06	0.07	0.02	0.02	0.02	0.05	**0.49**	0.11	0.08	0.05	0.01	0.1
L	0.12	0	0.02	0.02	0.06	0	0.21	0.01	**0.28**	0.01	**0.6**	0.15	0.04	0.02	0.16
M	0.03	0	0.09	0.04	**0.27**	0	0.01	0.01	0.02	0	**0.28**	0.02	0.01	0	0.03
N	0.07	**0.25**	0.1	0.02	0.01	0.04	0	0.04	0	0	0.03	**0.52**	0.04	0.02	0.04
X	0.06	0	0	0.02	0.2	0.04	0.01	0.03	0.09	0.02	0.2	0.06	0.2	**0.27**	0.03

Labels of subset 1 are displayed in rows, while labels of subset 2 are columns. Dice similarity coefficients over 0.25 are highlighted in bold.

## Discussion

In this exploratory study, our main aim was to test whether applying the currently available methods, which had been successfully used to explore resting-state networks in other species, yield interpretable results with our setup (proof-of-concept) and describe the spatial characteristics of these networks. To achieve this goal, we decided to use a data-driven method, which is not relying on a priori hypothesis regarding the supposed function of certain brain regions.

We found multiple, spatially distributed RSNs in dogs, the evolutionarily most distant taxa from humans scanned without anesthesia so far. To evaluate spatial overlap consistency, we calculated the Dice coefficients of the components from separate ICAs, which corroborated the robustness of the results. The localisation of these network correspond to the gross functional anatomical regions of the dog brain as described in the main neuroanatomical textbooks^32–35^ (e.g. primary visual field, auditory cortex, limbic circuit, sensorimotor region) and to the resting-state networks previously described in other species^7–10,12^, suggesting that our setup and analysis pipeline is suitable to detect resting-state networks in awake dogs.

Motion is still present in the data after standard pre-processing. The ICA method is able to deal with these structured noise effects via separating these in form of an additional (noise) ICA component^36^. The presence of such a component signals that the algorithm was successful at detecting and grouping the motion related fluctuations together and not a sign of problems with data quality. ICA is often used in human studies to remove the effects of residual motion as part of automated denoising pipelines^37^.

We described two visual network components (L & M), a finding similar to that of macaques^9^ and humans^38^, with one network corresponding to primary visual areas (medial visual cortical areas) and another encompassing visual association areas. Interestingly, Component M also shows a similar location to the pDMN component reported in the previous dog study^11^. In our study, we found two lateralized networks (Component D & E) including the striatum, amygdala, nucleus caudatus, cerebellum, insular cortex and thalamus, and a third, separate component covering the mid-cingulate gyrus (Component H). These regions are all nodes within the human saliency network^39^, and they may also play a similar role in dogs, but to determine the stability, repeatability and functional characteristics of this component(s), further studies are needed. Based on our findings, a necessary next step is to investigate the functional connectivity specifically between these putative saliency nodes on another dataset. We also found a bilateral frontal component, a finding similar to the results from praire voles^10^, marmosets^8^ and macaques^9^, but not reported outside of the superorder Euarchontoglires (primates and rodents) taxon so far.

Our results indicate that awake, unrestrained dogs’ possess a network (Component A) showing antero-posterior connectedness, containing areas from both the prefrontal cortex and the anterior cingulate cortex, with additional involvement of hippocampal regions. These regions correspond to regions indicated in the default mode network (DMN) described previously in humans^40^ and in animals^8,10^. However, the network we report here contains more regions than the traditional human DMN. For example, the composite gyrus, the hippocampi, splenial gyrus, and premotor area(although see^40^ on recent reports on the potential involvement of some of these additional regions in the human DMN). Therefore, it is problematic to label this component as DMN with only resting state ICA. In humans, specific regions of the DMN (MPFC, PCC and IPL) deactivate in response to a task. In order to definitively label the network we report here as the DMN, more research will need to show that analogous regions in the dog brain deactivate during a task. This dog network (Component A) does not include the parietal cortex. In humans, these parietal regions (angular gyrus, temporoparietal junction) are thought to be involved in complex cognitive processes such as accessing conceptual representations about events or items and theory of mind^40^, cognitive functions whose extent and level in dogs are debated^41^. Similarly to our findings, a putative DMN containing both the posterior cingulate cortex and frontal cortical areas have been desribed in rats^7^, voles^10^, ferrets^12^, marmosets^8^ and macaques^9^ so far. Our results do not support previous results in dogs^11^, which suggested dissociation of anterior and posterior regions of a DMN-like network, however our study diverged in several aspects which could account for the different findings. We utilized a larger sample size (4 vs. 22), longer data acquistion runs, different temporal filtering, lower dimensionality in the ICA. It is important to note that seed-based analysis (a model-driven approach) requires strong a priori hypothesis, a slight difference in the location of the spatial seed can have a significant impact on the spatial characteristics of the resulting RSNs^36^. Currently, we lack extensive fMRI studies regarding the functional properties of different dog brain regions, high resolution anatomical/cytological maps or molecular evidence to say which brain regions could be considered truly homologous e.g. how should be the dog cingulate gyrus divided into anterior, mid and posterior regions.

Dogs have some advantage regarding the scanning setup when compared to other model animals in fMRI studies. After special training, dogs are suitable to be scanned without sedation, which is beneficial as anaesthesia has a large impact on resting state functional connectivity^42^, and unlike other non-human animal species scanned awake, there is no need of mechanical restraints either, which also have the risk of influencing rs-network connectivity^43^.

In case of the relatively long TR which we used in our study, physiological artifacts can alias into the band of interest. For human adults, a normal resting heart rate is between 60 and 100 beats per minute (bpm). Reports of resting heart rate in dogs seem to show a large variation depending on the context in which they were measured. Veterinary visits or research procedures in case of laboratory dogs are stressful situations to dogs, and heart rates measured under these conditions reflects this. While the grand average of HR measured at veterinary visits^44^ and under laboratory conditions^45^ was around 124 beats/min, in another study, where dogs participated in a behavioural experiment with minimal physical activity, the grand average of HR was only around 80 beats/min^46^ and in a polysomnography study, where dogs were resting with their owners (even less physical activity), the grand average HR was only around 70 beats/min (Bálint *et al*.^47^). Respiration rate is indeed higher than in adults, but it is similar to the respiration rate of children (approx. 30/min)^48^. ICA is suited to deal with such physiological noise (e.g. respiration, pulsation), resulting in components with clusters mainly located in the white matter, cerebrospinal fluid and blood vessels (particularly arteries)^23^. Hence, we believe that regressing out signals from these regions via masking takes this into account, as these fluctuations related to physiological noise are also present in the white matter and cerebrospinal fluid space.

Our study is the first dog fMRI study utilizing atlas-based brain segmentation, mainly due to a lack of published brain masks delineating the limits of grey and white matter regions (while there is a binary atlas available, currently no probabilistic atlas exists). The creation, comparison and validation of such masks through different segmentation algorithms for dog brains is outside the scope of our current resting-state functional MRI study. Although we collected T1 weighted structural images from our subjects for the purpose of coregistration, the contrast of these images was not suitable to carry out reliable, individual segmentation based on intensity.

While our subjects did not look at a fixation cross during scanning, a study with adults, where the authors applied seed-based analysis showed no significant difference between eyes open and a fixation cross condition regarding intranetwork connectivity strengths^16^ and in another study on human children, where ICA results of a movie watching condition and a no stimuli resting condition were compared, revealed no significant differences in within-network connectivity between rest and movie watching^49^. In addition, resting state networks have been also successfully extracted from task-based co-activation patterns^50^. Based on these studies, it is unlikely that the presence of a static human during scanning would have significantly altered the spatial extent of the described resting-state networks.

The dog subjects are willingly motionless during scanning for an extended period of time (as performing a conscious action inhibiting their movements), a situation more closely resembling the conditions of human rs-fMRI measurements. While one may argue that the dogs are executing a rewarded “task” and not really resting, it is important to keep in mind that humans also consciously perform the same “hold still” task during fMRI measurments and most often do so in exchange to some predetermined reward (in form of financial compensation). Additionaly, while resting state networks are usually analysed in data collected during rest, they are also present when performing cognitive tasks^51^.

Based on the current study, we cannot provide information whether the described networks should be considered task positive or task negative (a dichotomy that has been recently challenged, see e.g.^52^) or regarding their functional characteristics (e.g. how is dogs’ visual network operating compared to visual networks of other species). The identification of the networks, like in other animal studies was based on the anatomical properties of the network, without investigating whether they show deactivation during the performance of specific tasks (as in anesthetized prairie voles^10^, rats^7^ and awake marmosets^8^). The description of these dog resting-state networks provide information for future studies to set up more specific a priori hypotheses to test the functional characteristics and interactions of the networks e.g. via also combining it with behavioural data.
